# Metabolic Profile of *Agropyron repens* (L.) P. Beauv. Rhizome Herbal Tea by HPLC-PDA-ESI-MS/MS Analysis

**DOI:** 10.3390/molecules27154962

**Published:** 2022-08-04

**Authors:** Martina Bortolami, Paola Di Matteo, Daniele Rocco, Marta Feroci, Rita Petrucci

**Affiliations:** 1 Department of Basic and Applied Sciences for Engineering, Sapienza University of Rome, Via del Castro Laurenziano 7, 00161 Rome, Italy; 2 Department of Ingegneria Meccanica ed Aerospaziale, Sapienza University of Rome, Via Eudossiana 18, 00184 Rome, Italy

**Keywords:** tryptophan, phenolic acids, flavonoids, antioxidants, ESI-MS fingerprinting, saccharides, amino acids, small organic acids

## Abstract

*Agropyron repens* (L.) P. Beauv. (couch grass) is a world-wide infesting rhizomatous plant with pharmacological applications. Chemical research is focused on its allelopathic and anti-inflammatory components, which are mainly present in the essential oil. Conversely, the aqueous extracts have been sparingly investigated, although the herbal tea is by far the most used formulation. To fill the gap, the metabolic profile of *Agropyron repens* rhizome herbal tea was investigated by electrospray ionization (ESI) tandem–mass spectrometry (MS/MS); the phenolic profile was investigated by HPLC-PDA-ESI-MS/MS. ESI-MS fingerprinting was provided, evidencing diagnostic ions for saccharides, organic acids and amino acids. The HPLC-PDA-ESI-MS/MS analysis evidenced at least 20 characteristic phenolic compounds, the most representative being caffeoyl and feruloyl quinic esters, followed by coumaric, caffeic and ferulic acids, and hesperidin among flavonoids. In addition, the essential amino acid tryptophan was identified for the first time. The results suggest new perspectives of applications for *Agropyron repens* rhizome.

## 1. Introduction

*Agropyron repens* (L.) P. Beauv. or *Elymus repens* (L.) Gould (couch grass, quackgrass) is a well-known perennial rhizomatous plant, native from Europe and Central Asia, but widespread in the world. Its notoriety is mainly due to its infesting action in crops [1] and to a quite minor extent of its use as a traditional medicinal plant for urinary problems [2,3,4]. The infesting action is due to its allelopathic effects [5] on crops. It is an aggressive competitor for many plants. Moreover, allelopathic toxins are produced from plant decaying residues. However, the traditional medicinal use in urinary calculus disease has received scientific confirmation, including for its other pharmacological effects [6]. Among the pharmacological actions, there are the hypoglycemic [7], hypolipidemic [8], anti-inflammatory [9,10], and antidiabetic activities [11], effects on motility [12], and beneficial effects in urinary tract infections [13].

For these reasons, the chemical research has mainly focused on the allelopathic compounds and on the anti-inflammatory molecules, primarily found in the essential oil (volatiles, 0.01–0.05% of the plant mass by distillation). In particular, agropyren (possibly 1-phenylhex-2-en-4-yne, C_12_H_12_) was thought to be one of the active molecules (and thus named after *Agropyron repens*) [14], but its presence in the essential oil was not confirmed by later studies [15,16]. Aqueous extracts were rarely characterized, and seldom a quantitation was reported, while organic extracts as well as alcoholic and hydro-alcoholic extracts were characterized, and in some cases, a quantitation of active compounds was carried out [17,18,19,20,21,22,23,24,25,26,27,28]. Early reports failed in the identification of allelopathic compounds [17,18], while Weston in 1987 identified tricin (5,7,4′-trihydroxy-3′,5′-dimethoxyflavone) as an allelopathic molecule in water extracts [19]. Later, other allelopathic molecules were identified: 6-hydroxy-1,2,3,4-tetrahydro-β-carboline-3-carboxylic acid (6-HTβC-3-COOH, toxic towards slug species) from hydro-alcoholic extracts [20], (*E*)- and (*Z*)-16-hydroxyhexadecyl esters of *p*-hydroxycinnamic acid (mono- and diesters) from organic extracts [21,22], also with antiadhesive activity against uropathogenic *E. coli* [23], 5-*n*-alkylresorcinols from acetone extracts [24], 3-indoleacetic acid (IAA, plant growth hormone and inhibitor) from hydro-alcoholic extracts [25], 2,4-dihydroxy-2*H*-1,4-benzoxazin-3-one (DIBOA, phytotoxin), 2,4-dihydroxy-7-methoxy-2*H*-1,4-benzoxazin-3-one (DIMBOA, phytotoxin) and 2-hydroxy-1,4-benzoxazin-3-one (HBOA, phytotoxin) from water extracts [26,27], 5-hydroxyindole-3-acetic acid (5-HIAA, growth hormone) and 5-hydroxytryptophan (5-HTP, growth inhibitor) from alcoholic extracts [28]. 5-HTP is a natural molecule, also used as a drug [29], of particular importance for humans. It is obtained from tryptophan (TP) by enzymatic reaction, and its decarboxylation produces serotonin (5-hydroxytryptamine, 5-HT), an important neurotransmitter. Moreover, 5-HT can be transformed into melatonin (*N*-acetyl-5-methoxytryptamine), an important hormone.

Tryptophan is one of the essential amino acids that cannot be synthesized by humans and must be supplied with food. Besides its role of an amino acid in peptide synthesis and its importance as a precursor of 5-HTP and derived compounds, it is a precursor of niacin (vitamin B3) and auxins [30,31]. Tryptophan is present in various vegetals (soy, rice, corn, cotton, etc.) [32], but no paper reports the presence of tryptophan in *Agropyron repens* rhyzome (to the best of our knowledge).

The *Agropyron repens* components were recently reviewed by the European Medicine Agency [33], and the literature data are summarized in Table 1 [33,34], along with the relevant bibliography [5,6,10,11,16,19,35,36,37,38,39,40,41].

There is thus a lack of information on the real composition of *Agropyron repens* herbal tea, the most used formulation in traditional medicine and herbalism. In the use of the infusion for therapeutic purposes, not only are the active ingredients ingested, but everything that from the dry rhizome is extracted with water.

For this reason, since the herbal tea sees only water as a solvent, we focused on *Agropyron repens* rhizome aqueous extracts at three different temperatures: 25 (room temperature), 50 and 100 °C (boiling water, herbal tea normal infusion). We decided to investigate 25 and 50 °C extracts in order to understand the role of temperature on extract constituents.

## 2. Results and Discussion

Three independent commercial samples of *Agropyron repens* rhizome, named AR1, AR2, AR3, were used to prepare the herbal tea in Milli-Q water brought to a boil, according to the dose and procedure recommended by the Official Pharmacopoeia of the Italian Republic, as reported below in Materials and Methods, with the aim to evaluate the metabolic profile and the individual phenolic compounds content that consumers may take with a cup of tisane. The rhizome samples were also extracted with water at room temperature (25 °C) and at 50 °C to evaluate the effect of temperature. The extracts were first analyzed by tandem mass spectrometry (MS/MS) by direct infusion into the ESI source, with the aim to explore the matrix as a whole.

### 2.1. Untargeted ESI-MS/MS Profile

**Figure 1:**
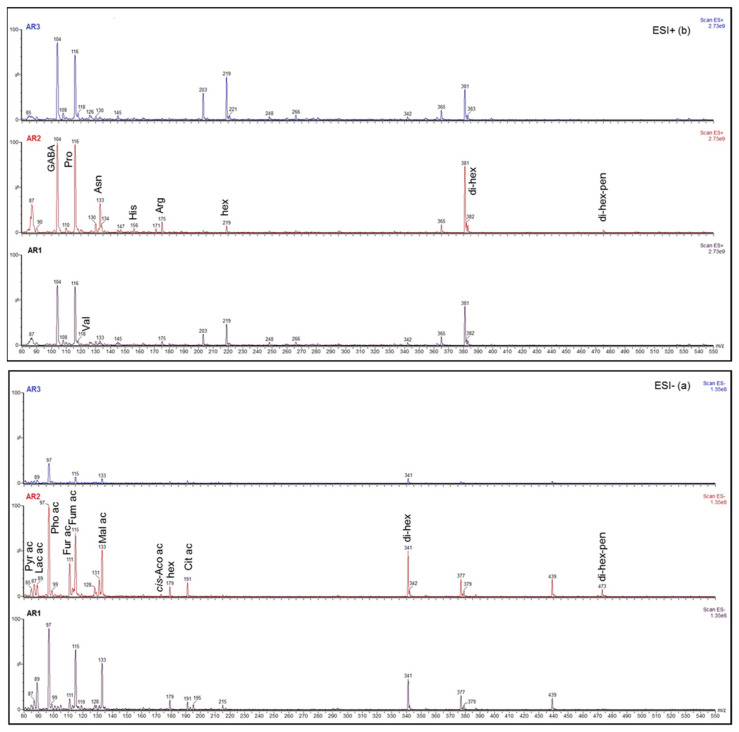
ESI-MS/MS profile of rhizome *Agropyron repens* herbal tea, samples AR1, AR2, AR3, in negative ionization ESI- (**a**) and positive ionization ESI+ (**b**), with signals normalized to the highest one for a direct comparison among samples. (**a**) Pyr ac (pyruvic acid, [M-H]^−^ = 87 *m*/*z*); Lac ac (lactic acid, [M-H]^−^ = 89 *m*/*z*); Pho ac (phosphoric acid, [M-H]^−^ = 97 *m*/*z*); Fur ac (2-furoic acid, [M-H]^−^ = 111 *m*/*z*); Fum ac (fumaric acid, [M-H]^−^ = 115 *m*/*z*); Mal ac (malic acid, [M-H]^−^ = 133 *m*/*z*); *cis*-Aco ac (*cis*-aconitic acid, [M-H]^−^ = 173 *m*/*z*); hex (hexose, [M-H]^−^ = 179 *m*/*z*); Cit ac (citric acid, [M-H]^−^ = 191 *m*/*z*); di-hex (di-hexose, [M-H]^−^ = 341 *m*/*z*, [M+Cl]^−^ = 377 *m*/*z*, [M+Cl+2]^−^ = 379 *m*/*z*); di-hex-pen (di-hexose-pentose, [M-H]^−^ = 473 *m*/*z*). (**b**) GABA (γ-aminobutyric acid, [M+H]^+^ = 104 *m*/*z*); Pro (proline, [M+H]^+^ = 116 *m*/*z*); Val (valine, [M+H]^+^ = 118 *m*/*z*); Asn (asparagine, [M+H]^+^ = 133 *m*/*z*); His (histidine, [M+H]^+^ = 156 *m*/*z*); Arg (arginine, [M+H]^+^ = 175 *m*/*z*); hex ([M+Na]^+^ = 203 *m*/*z*, [M+K]^+^ = 219 *m*/*z*); di-hex ([M+Na]^+^ = 365 *m*/*z*, [M+K]^+^ = 381 *m*/*z*); di-hex-pen ([M+H]^+^ = 475 *m*/*z*, [M+Na]^+^ = 497 *m*/*z*, [M+K]^+^ = 513 *m*/*z*).

The untargeted analysis of the extracts evidenced quite similar profiles, regardless of type of sample and extraction temperature. The ESI-MS/MS metabolic profile of the *Agropyron repens* rhizome herbal tea is shown in Figure 1, in both ESI- (*a*) and ESI+ (*b*), in which the profiles of AR1, AR2 and AR3, from bottom to top, respectively, were reported.

No signals attributable to phenolic compounds were evidenced. However, the phenolic fraction in natural sources is generally low compared to other fractions such as organic acids or sugars; therefore, the phenolic signals might be expected to be hidden by the others in the direct infusion experiments. Conversely, three sets of well-evidenced signals were ascribed to saccharides, small organic acids and amino acids, based on *m*/*z* values relative to deprotonated/protonated ions [M-H]^−^/[M+H]^+^, sodium, potassium and chloride adducts [M+Na]^+^, [M+K]^+^ and [M+Cl]^−^, respectively, and fragmentation spectra, obtained in daughter scans by selecting each precursor ion.

#### 2.1.1. Saccharides

A di-hexose C_12_H_22_O_11_ with M = 342 Da was suggested by signals in both ESI- and ESI+; (a) and (b) in Figure 1, respectively. The assignment was supported by the ESI- signals at *m*/*z* 341, 377 and 379, relative to the anions [M-H]^−^, [M+Cl]^−^ and [M+Cl+2]^−^, respectively, and by the ESI+ signals at *m*/*z* 365 and 381, relative to [M+Na]^+^ and [M+K]^+^, respectively, in good agreement with data reported in the literature for the di-hexoses maltose [42,43], and sucrose [44]. The ESI- fragmentation spectrum of [M-H]^−^ = 341 *m*/*z* evidenced fragments at *m*/*z* 179 and 161, deriving from the scission of the glycoside bond into the hexose (hex) units, and 149, 119, 89 and 71 as hexose fragments, in agreement with those reported for D-fructose [45]. The ESI+ fragmentation spectrum of [M+K]^+^ = 381 *m*/*z* evidenced fragments at *m*/*z* 219 and 201, corresponding to [M(hex)+K]^+^ and [M(hex)+K-H_2_O]^+^, respectively; analogously, the fragmentation spectrum of [M+Na]^+^ = 365 *m*/*z* evidenced the ions [M(hex)+Na]^+^ and [M(hex)+Na-H_2_O]^+^ at *m*/*z* 203 and 185, respectively. These data agree with literature for sucrose [44].

A di-hexose–pentose trisaccharide C_17_H_30_O_15_ with M = 474 Da was suggested by signals in both ESI- and ESI+; (a) and (b) in Figure 1, respectively. The assignment was supported by signals at *m*/*z* 473, 475, 497 and 513, relative to the ions [M-H]^−^, [M+H]^+^, [M+Na]^+^ and [M+K]^+^, respectively. The ESI- fragmentation spectrum of [M-H]^−^ = 473 *m*/*z* evidenced fragments at *m*/*z* 341 and 131, in agreement with the glycoside bond scission leading to the disaccharide discussed above and a pentose residual unit (Δ = 132 Da) [45,46].

Lastly, the presence of free hexoses with M = 180 Da was suggested by signals in both ESI- and ESI+; (a) and (b) in Figure 1, respectively. The assignment was supported by the signal [M-H]^−^ = 179 *m*/*z*, giving the same fragments discussed above when it was selected as a precursor ion, and signals at *m*/*z* 203 and 219, relative to [M+Na]^+^ and [M+K]^+^, respectively, reported in the literature for glucose [47].

The di-hexose and free hexoses were well evidenced in all samples, with a different distribution: the hexose/di-hexose signal ratio was in the order AR2 < AR1 < AR3. The di-hexose–pentose was well characterized, albeit weak, in AR2, while a low signal was evidenced in AR1 and AR3. Since a similar profile was observed for extracts at room temperature (25 °C), these mono-, di- and tri-saccharides do not derive from any hydrolysis due to temperature, but they must be present as such in the dry rhizomes. Differences among AR1, AR2 and AR3 might be due to different geographical origin, collection period, drying process and/or storage of the rhizomes, information not available to consumers. Slight differences among the samples were also observed regarding humidity, water activity and microbiological analysis, important data regarding food safety and quality. As shown in Table 2, all samples were below the threshold values recommended by the World Health Organization (TMC < 10^7^ CFU/g and Y/M < 10^5^ cfu/g) [48,49,50]. Differences in the TAC values could be attributed to the microbial contamination that may occur during the product preparation from the harvesting to packaging, handling, dispensing and storage. The microbiological data were in agreement with water activity results, shown in Table 2, that were below the limit for all the samples. Water activity indicates the unbound water available to microorganisms for their growth: high values of water activity (0.90) inhibit the growth of most microorganisms: for yeasts and molds and for all microorganisms, the limit values are 0.70 and 0.60, respectively.

Compared with the literature, the mono-, di- and tri-saccharides described above might be free fructose and glucose, sucrose and di-hexose-xylose, respectively, taking into account what is reported in the literature for triticin, the *Agropyron repens* characteristic polysaccharide fructosan [33]. Triticin is mainly composed of fructose with terminal glucose residue linked as a sucrose unit, at least according to the hydrolysis products reported in hot or boiling water [35]; furthermore, fructose, glucose and a disaccharide, hydrolizing mainly to glucose and fructose with xylose, were reported for aqueous alcohol extracts [35]. Fructose has the lowest glycemic index compared with some widespread carbohydrate-containing foods [51]. A further dedicated analysis is needed for definitively elucidating the structures of the di-hexose and the di-hexose-pentose.

#### 2.1.2. Small Organic Acids

Among the ESI- signals at low mass values, eight small organic acids were suggested by the ESI- signals at *m*/*z* 87, 89, 97, 111, 115, 133, 173 and 191, likely corresponding to the deprotonated forms [M-H]^−^ of pyruvic, lactic, phosphoric, 2-furoic, fumaric, malic, *cis*-aconitic and citric acids, respectively (Figure 1a). The assignments were supported by the absence of the corresponding [M+H]^+^ or adducts in ESI+ (Figure 1b), and by fragmentation spectra obtained in daughter scans by selecting the precursor ion [M-H]^−^, besides a good agreement with the literature [52,53,54,55,56]. Data are shown in Table 3.

Phosphoric, fumaric and malic acids were prevalent in all samples; pyruvic and lactic acids were differently distributed in the samples; 2-furoic acid was more abundant in AR2; *cis*-aconitic acid was found only in AR2. To the best of our knowledge, among these, only maleic and citric acids were reported in ethyl acetate extracts from *Agropyron repens* shoot and root exudates [6], along with *trans*-aconitic acid, for which a different fragmentation spectrum with respect to *cis*-aconitic acid discussed above was reported in the literature [57].

#### 2.1.3. Amino Acids

Among the ESI+ signals at low mass values, six amino acids were suggested by the ESI+ signals at *m*/*z* 104, 116, 118, 133, 156 and 175, likely corresponding to the protonated forms [M+H]^+^ of *γ*-aminobutyric acid (GABA), proline, valine, asparagine, histidine and arginine (Figure 1b). The assignments were supported by the absence of the corresponding anions [M-H]^−^ in ESI- (Figure 1a) and by fragmentation spectra, obtained in daughter scans by selecting each precursor ion [M+H]^+^, besides a good agreement with the literature [58,59,60]. Data are shown in Table 4.

GABA and proline were prevalent in all samples. Valine was better evidenced in AR1 and AR3, while AR2 was in general richer in asparagine, histidine and arginine. Differences in amino acids composition might be ascribed to different collection periods and/or storage [60].

All masses discussed above (saccharides, small organic acids, amino acids) were evidenced as not retained compounds in the full scan chromatogram by HPLC-ESI-MS/MS analysis, within the first 2–3 min elution, in agreement with the high polarity of compounds such as sugars, small organic acids and amino acids.

On the whole, a similar behavior was observed for the three samples AR1, AR2 and AR3 with regard to extraction temperature. Extraction time being equal (2 h), slight differences were observed between 25 and 50 °C: a weak increase to no effect was observed on sugar and amino acid signals at 50 °C, and a slight increase was observed for organic acid signals at 50 °C. Since weak differences were observed regarding the sugar signals in the herbal tea (100 °C, 10 min extraction), with respect to 25 and 50 °C extracts, the extraction time seemed to affect the sugar extraction more than temperature. Conversely, a strong increase was generally observed regarding organic acids signals, and a decrease was observed for amino acid signals, mainly for GABA. Temperature therefore seemed to affect the extraction of organic acids and amino acids more than extraction time.

The ESI mass spectral fingerprinting of *Agropyron repens* rhizome herbal tea is here provided for the first time, at least to the best of our knowledge, with useful application as fast and simple quality control or fraud (the three independent samples showed similar profiles).

### 2.2. HPLC-PDA-ESI-MS/MS Targeted Analysis

**Figure 2:**
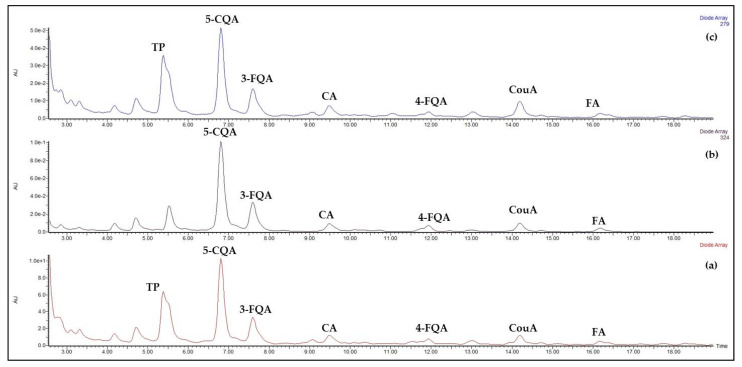
AR2 herbal tea diluted 1:10 with mobile phase: (**a**) PDA chromatogram; (**b**) EC at λ = 324 nm; (**c**) EC at λ = 279 nm. TP: tryptophan; 5-CQA: 5-caffeoylquinic acid; 3-FQA: 3-feruloylquinic acid; CA: caffeic acid; 4-FQA: 4-feruloylquinic acid; CouA: coumaric acid; FA: ferulic acid.

The HPLC-PDA chromatograms of the aqueous extracts results were quite similar, regardless of the type of sample and extraction temperature, at least from a qualitative point of view. A few abundant peaks were evidenced at low elution times, within the first 18 min, with characteristic absorption at λ 322–325 nm, and one peak was observed with characteristic absorption at 279 nm. The PDA chromatogram of AR2 herbal tea, as an example, is shown in Figure 2a, along with the extracted chromatograms (EC) at 324 nm (b) and 279 nm (c).

**Scheme 1:**
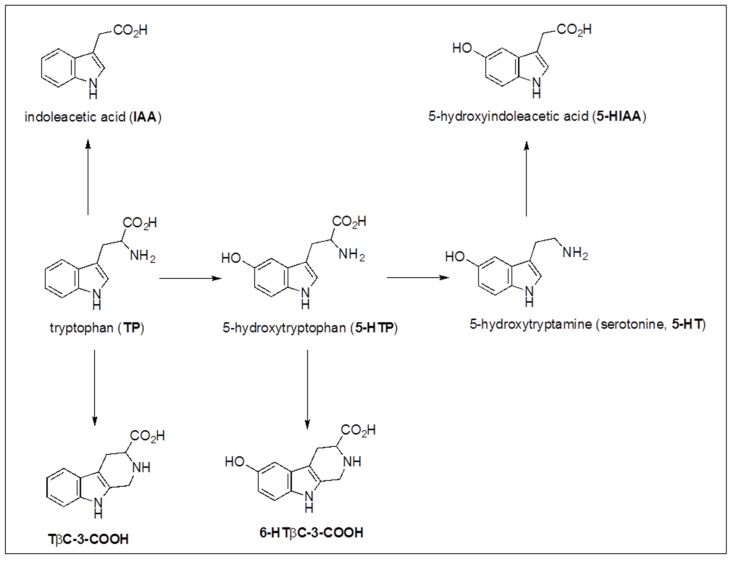
Biosynthetic pathways of growth hormones and allelopathic molecules starting from tryptophan (TP).

The absorptions at 322–325 nm are characteristic of hydroxycinnamic acids (HCAs) and their quinic esters (CQAs) that were confirmed by the targeted analysis of phenolic compounds (vide infra), while the peak absorbing at 279 nm and retention time t_R_ = 5.51 min were identified as tryptophan (TP) by comparing retention time t_R_, UV–Vis spectrum, ESI+, ESI- and fragmentation spectra with a standard sample. Although TP has been found in various vegetals [32], no paper reports its presence in *Agropyron repens* rhizome, at least to the best of our knowledge. Since it was found in all samples including extracts at room temperature, the possibility that it might have been generated from any hydrolytic process seemed to be excluded. TP is the precursor of various bioactive compounds, as growth hormones and allelopathic molecules, i.e., IAA, 5-HIAA, 5-HTP, TβC-3-COOH and 6-HTβC-3-COOH (see Scheme 1), were reported mainly in alcoholic extracts of *Agropyron repens* rhizome [20,25,28]. The presence of these compounds in the herbal tea was investigated by selecting the corresponding *m*/*z* monoisotopic values for the ions [M-H]^−^ in dedicated chromatographic runs: 174 for IAA, 190 for 5-HIAA, 219 for 5-HTP, 215 for TβC-3-COOH, and 231 for 6-HTβC-3-COOH.

**Figure 3:**
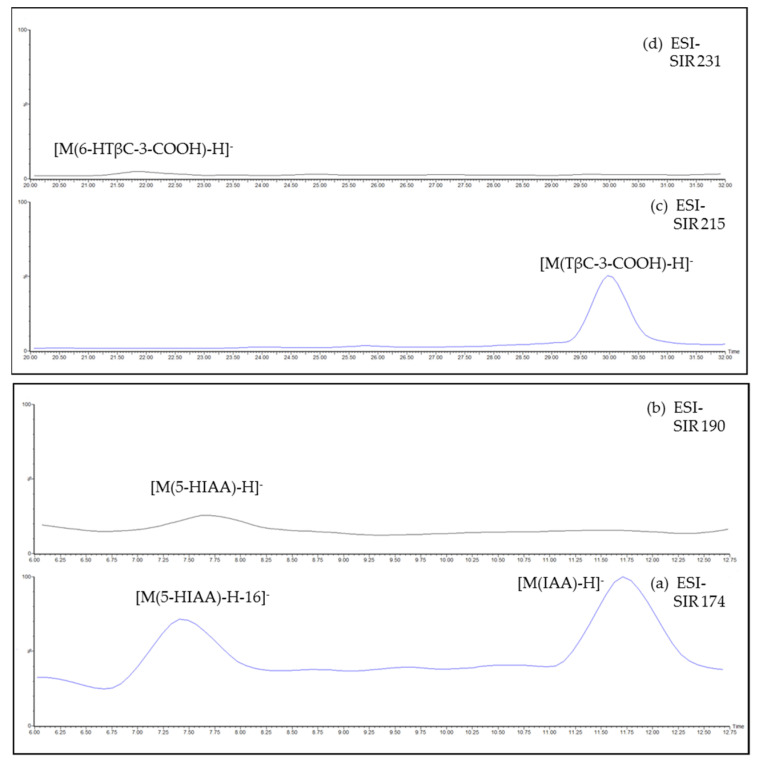
AR3 herbal tea, diluted 1:10 with mobile phase, ESI-SIR channels at *m*/*z* 174 (**a**), 190 (**b**), 215 (**c**) and 231 (**d**), corresponding to the ion [M-H]^−^ of IAA (**a**), 5-HIAA (**b**), TβC-3-COOH (**c**) and 6-HTβC-3-COOH (**d**).

No signal was evidenced in the SIR channel 219; therefore, the presence of 5-HTP was excluded. Conversely, peaks evidenced in the SIR channels 174, 190, 215 and 231 might support the presence of IAA, 5-HIAA, TβC-3-COOH and 6-HTβC-3-COOH, although in low amounts, as shown in Figure 3a–d, from bottom to top, respectively, in which AR3 herbal tea SIR (selected ion recording) chromatograms are reported as an example.

5-HIAA seemed to be further supported by the fragment [M(5-HIAA)-H-16]^−^ = 174 *m*/*z* evidenced in the SIR channel 174 (Figure 3a). The elution order, 7.70 min (5-HIAA), 11.71 min (IAA), 21.69 min (6-HTβC-3-COOH) and 29.97 min (TβC-3-COOH), was consistent with the structures (see Scheme 1), but it is obvious that further investigation with comparing standards will be needed to confirm their presence. However, no interference of these compounds on human health has been reported in the literature, at least to the best of our knowledge [20].

Considering that TP is an essential amino acid and precursor of key biomolecules important for humans, such as 5-HTP and serotonin (5-HT), new perspectives of applications might arise for *Agropyron repens* rhizome.

**Scheme 2:**
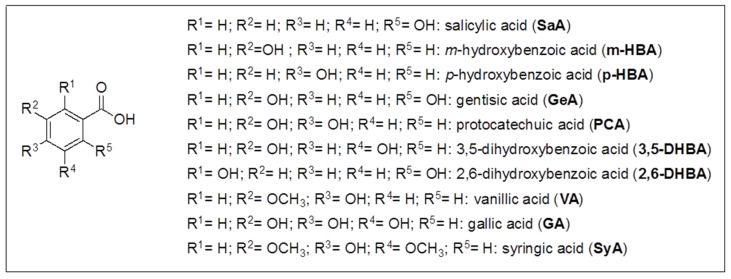
Hydroxybenzoic acids (HBAs) structures.

**Scheme 3:**
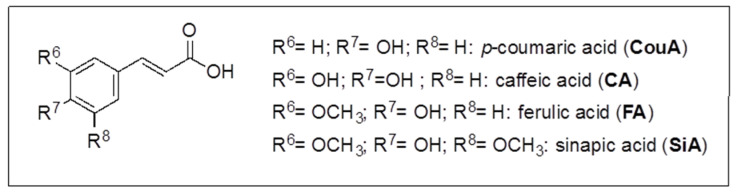
Hydroxycinnamic acids (HCAs) structures.

**Scheme 4:**
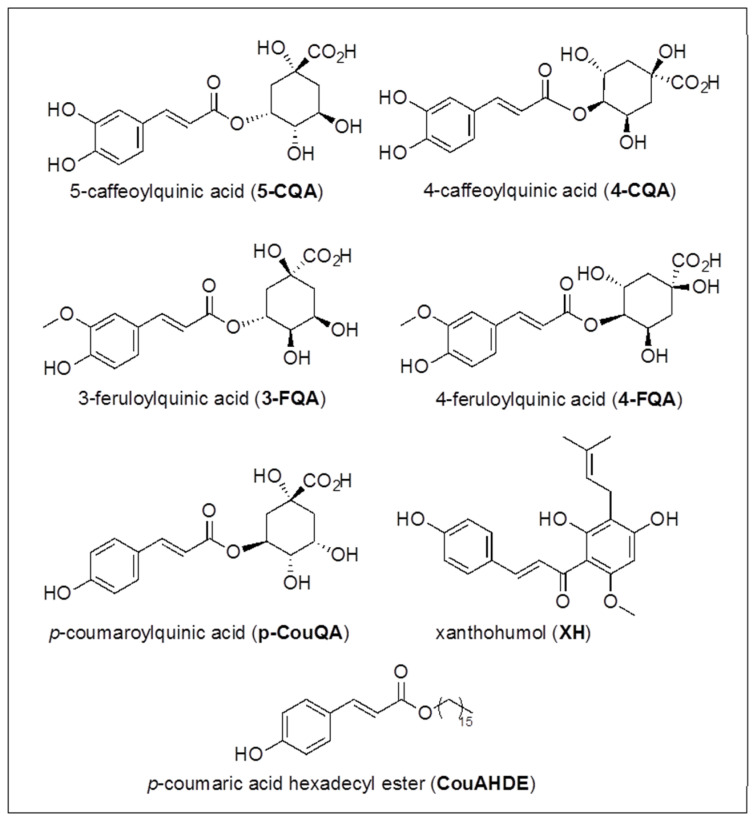
Hydroxycinnamic acids derivatives structures.

**Scheme 5:**
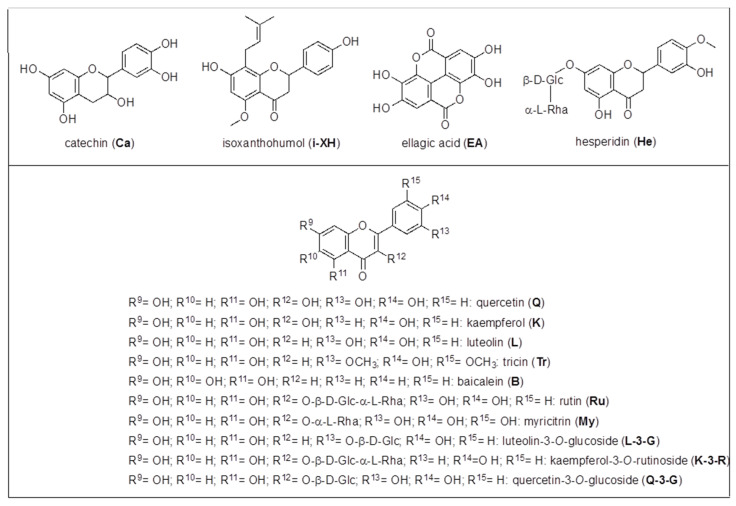
Flavonoids (Fs) structures.

Some of the other chromatographic peaks evidenced in Figure 2 were assigned, and quantitated in most cases, by HPLC-ESI-MS/MS SIR mode analysis, carried out with a method previously developed [43,61,62] and briefly described in Materials and Methods, for the identification and quantitation of 26 phenolic compounds including ten hydroxybenzoic acids (HBAs), four hydroxycinnamic acids (HCAs), two quinic esters of caffeic acid (CQAs), eight flavonoids and two prenylflavonoids (Fs). In detail, HBAs: *p*-hydroxybenzoic acid (*p*-HBA), *m*-hydroxybenzoic acid (*m*-HBA), *o*-hydroxybenzoic acid (salicylic acid, SaA), 3,5-dihydroxybenzoic acid (3,5-DHBA), 3,4-dihydroxybenzoic acid (protocatechuic acid, PCA), 2,5-dihydroxybenzoic acid (gentisic acid, GeA), 2,6-dihydroxybenzoic acid (2,6-DHBA), 3,4,5-trihydroxybenzoic acid (gallic acid, GA), vanillic acid (VA), syringic acid (SyA) (structures in Scheme 2); HCAs: caffeic acid (CA), *p*-coumaric acid (CouA), sinapic acid (SiA), ferulic acid (FA) (structures in Scheme 3); CQAs: 4-caffeoylquinic acid (4-CQA), 5-caffeoylquinic acid (5-CQA) (structures in Scheme 4); Fs: catechin (Ca), quercetin (Q), kampferol (K), rutin (Ru), myricitrin (My), quercetin-3-*O*-glucoside (Q-3-G), kaempferol-3-*O*-rutinoside (K-3-R), hesperidin (He), isoxanthohumol (i-XH) (structures in Scheme 5) and xanthohumol (XH) (structure in Scheme 4).

TP was quantitated with the same chromatographic run by using the calibration curve y = 34379x − 1228.3, R^2^ = 0.9995, obtained as described in Materials and Methods, with LOD = 2.64 μg/mL and LOQ = 8.00 μg/mL.

In addition, ellagic acid (EA) (structure in Scheme 5) was identified by comparison with standard and was quantitated as Q content by using the quercetin calibration curve [43]. Lastly, 3-feruloylquinic acid (3-FQA) and 4-feruloylquinic acid (4-FQA) (structures in Scheme 4) were tentatively assigned, supported by chromatographic data, UV–Vis spectral data and mass fragmentation spectra, in strong agreement with the literature [63], and quantitated as FA content by using the ferulic acid calibration curve [43].

The presence of CouA quinic derivative (*p*-CouQA, structure in Scheme 4) was also investigated by SIR analysis of the ion [M-H]^−^ = 337 *m*/*z*, but the acquired mass spectral and chromatographic data results were insufficient to any conclusion. All data are shown in Table 5, in which the amounts are reported in μg/g of dry sample of rhizome, as mean values ± standard deviation.

Quite similar phenolic profiles were found for the three samples of AR1, AR2 and AR3 herbal tea. Among the 26 analyzed phenolic compounds, five of them, i.e., 3,5-DHBA, m-HBA, Ca, My and Ru, were not detected in any samples, and three of them, i.e., EA, Q and K, were detected in all samples with content under LOD, 70 and 60 μg/L of extract, respectively [43]. Furthermore, similar contents of GA, PCA, VA, SyA, SiA, SaA and i-XH were found for AR1 and AR2, while AR3 was found to be significantly different (*p* < 0.05) for the same compounds, except for i-XH, which was not detected.

Conversely, similar contents of CA, FA and 2,6-DHBA were found for AR2 and AR3, while AR1 was found to be significantly different (*p* < 0.05) for the same compounds. The three sample results were significantly different (*p* < 0.05) for GeA, *p*-HBA, CouA, 4-CQA, 5-CQA, 3-FQA, 4-FQA and He, except for TP. Q-3-G and K-3-R were identified only in AR2 in amounts under LOD. XH was identified only in AR1 and AR2, in amounts under LOD.

Despite some differences regarding the individual amount of the detected compounds, likely due to geographical origin, collection period, drying process and/or storage of the AR1, AR2 and AR3 rhizomes, analogously to what is discussed above, a well-defined phenolic profile was found for the *Agropyron repens* herbal tea, mainly composed of HCAs, in their free forms and as ester derivatives of quinic acid. In particular, 5-CQA was the most abundant in all samples (57.40–112.22 μg/g), followed by 4-CQA (32.43–65.66 μg/g), 3-FQA (9.99–53.99 μg/g), 4-FQA (3.97–27.14 μg/g), CouA (5.83–8.53 μg/g), CA (2.55–6.98 μg/g) and to a lesser extent FA (1.97–4.05 μg/g) and SiA (0.40–0.78 μg/g) (Table 5; Figure 2a,b). Lower but still significant amounts were found for HBAs, among which *p*-HBA was the most abundant (3.64–9.52 μg/g), followed by VA (3.18–5.22 μg/g), PCA (1.21–7.16 μg/g), SyA (1.89–3.69 μg/g), and to a lesser extent GA (0.67–2.80 μg/g), GeA (0.86–2.07 μg/g) and SaA (0.29–1.37 μg/g). Among the investigated flavonoids, only He was quantitated (1.37–4.40 μg/g). With regard to the prenylflavonoid i-XH and its precursor XH, they were detected only in AR1 and AR2, and in both cases, XH was found in lower amounts (<LOD, 1.13 μg/L) than i-XH. Taking into account that i-XH generally generates from XH by heating [64], these data seemed consistent with some rhizome drying processes.

TP was found in the herbal tea among the most abundant compounds, with quite similar content in AR1 (19.92 μg/g) and AR3 (13.01 μg/g) and in high levels in AR2 (203.66 μg/g), a trend confirmed in the extracts at different temperatures. Such a difference might be ascribed to different origin and/or treatment of the three samples of rhizome; however, all data confirm the presence of TP as a free amino acid, which was unexpected data since it was never reported before in *Agropyron repens*.

The statistical analysis carried out for each compound quantitated in the three different extracts (herbal tea, 2 h stirring at 25 °C and 2 h stirring at 50 °C), evidenced different behaviors for different compounds: weak effects were evidenced for some molecules, temperature/extraction time combined effects were observed for others. In general, the herbal tea resulted in a good compromise of temperature (100 °C) and extraction time (10 min), providing a total of extracted phenolic antioxidants in the range of 126.89–289.09 μg/g of rhizome, with TP as an added value.

**Scheme 6:**
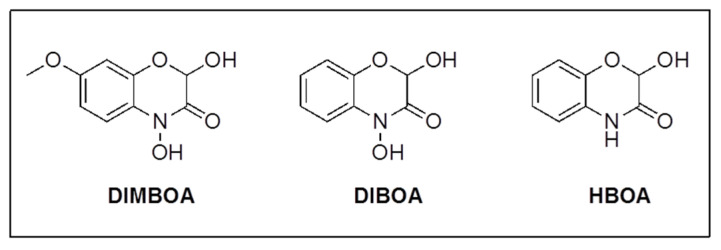
Allelopathic molecules DIMBOA, DIBOA and HBOA structures.

Conversely, the presence of some phytotoxins in the herbal tea cannot be excluded. The presence of DIBOA, DIMBOA and HBOA (structures in Scheme 6), reported as allelopathic constituents of ethyl acetate extracts from some parts of the plant [6], was investigated in the herbal tea as well in the aqueous extracts at different temperatures by selecting the corresponding *m*/*z* monoisotopic values for the ion [M-H]^−^ in dedicated chromatographic runs: 180 for DIBOA, 210 for DIMBOA and 164 for HBOA.

**Figure 4:**
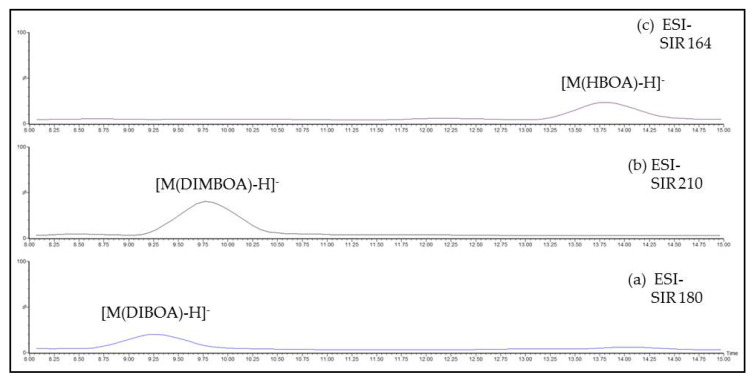
ESI- SIR channels at (**a**) 180, (**b**) 210 and (**c**) 164 *m*/*z*, corresponding to the ion [M-H]^−^ of (**a**) DIBOA, (**b**) DIMBOA and (**c**) HBOA, respectively. AR3 herbal tea as an example.

Three chromatographic peaks, each one in the corresponding SIR channel, were observed at 9.21, 9.77 and 13.76 min, as shown in Figure 4a–c, respectively, in which data for AR3 herbal tea are reported as an example. Besides the molecular mass, chromatographic data as t_R_ and elution order seemed in good agreement with the structures of DIBOA, DIMBOA and HBOA (see Scheme 6).

Lastly, the presence of the flavonoids luteolin, as free form (L) and as glucoside derivative (L-3-G), baicalein (B) and tricin (Tr) (structures in Scheme 5), and the hexadecyl coumaric acid ester (CouAHDE) (structure in Scheme 4), reported in the literature as constituents of *Agropyron repens* rhizome extracts [10,19,21,22,38], were investigated by the SIR technique, selecting in independent channels the monoisotopic ion [M-H]^−^ *m*/*z* values 285 for L, 447 for L-3-G, 269 for B, 329 for Tr and 387 for CouAHDE. On the basis of the molecular mass values and chromatographic data, the presence of luteolin was excluded, in the free form as well as for the glucoside derivative. Analogously, the presence of CouAHDE was excluded, as expected due to its low polarity. Ambiguous data were found for baicalein, whose presence cannot be supported. Conversely, a well-defined peak with [M-H]^−^ = 329 *m*/*z* at t_R_ = 31.07 min suggested the presence of Tr, the elution time being consistent with the structure.

If the absence of a peak in the SIR channel may exclude the presence of any isobaric compounds with that of the *m*/*z* value, including the searched one, the presence of a peak in the SIR channel needs further investigation to definitively assign it to a target molecule, which cannot be excluded until then.

## 3. Materials and Methods

### 3.1. Chemicals and Solvents

All chemicals were of analytical grade, purchased from Sigma-Aldrich (Milano, Italy) and used as received. HPLC-grade acetonitrile and methanol were purchased from Carlo Erba (Milano, Italy); HPLC-grade water was freshly prepared with the Milli-Q purification system (Millipore, Vimodrone, Italy).

### 3.2. Plant Material Characterization

Three commercial samples of rhizomes of *Agropyron repens* (L.) P. Beauv. (Quackgrass), marked above as AR1, AR2 and AR3, were purchased from three different local herbal medicine shops; each sample was characterized by dealer name, batch number and expiration date. The samples, stored under vacuum, are kept at our laboratory for further reference. Water activity and absolute humidity of each sample were measured (in duplicate) before use, by a Schaller Humimeter Rh2. The microbiological analysis was performed on 5.00 g of each sample aseptically removed from the shop package, placed in a stomacher bag, diluted with 0.9% NaCl solution and homogenized with a Stomacher LAB Blender 400 (Pbi International); the total aerobic count was determined on 3M^®^ Petrifilm aerobic total count, after incubation at 30 °C for 24–48 h; yeasts and molds were detected on 3M^®^ Petrifilm yeast and molds at 30 °C for 24–72 h; lactic acid bacteria were enumerated on 3M^®^ Petrifilm lactic acid bacteria incubated at 37 °C for 24–72 h; Enterobacteriaceae were counted on 3M^®^ Petrifilm Enterobacteriaceae at 37 °C for 24 ± 2 h. All measurements were performed in duplicate.

### 3.3. Agropyron repens (*L.*) P. Beauv. Rhizome Extraction Procedure

The rhizome herbal tea was obtained according to dose and procedure recommended by the Official Pharmacopoeia of the Italian Republic, by adding 1.00 g di AR1, AR2 and AR3, in turn, to 10 mL of Milli-Q purified water previously brought to boil. After 10 min, the mixture was filtered at 0.22 μm, brought back to room temperature, appropriately diluted (1:10, 1:100, 1:200) with the mobile phase (A:B, 95:5, *v*/*v*, vide infra) and injected (20 μL) in triplicate for the analysis by HPLC-PDA-ESI-MS/MS.

Aqueous extracts (25 or 50 °C) were obtained by adding 10 mL of Milli-Q-purified water to 1.00 g of AR1, AR2 and AR3, in turn, and keeping the mixtures at room temperature or at 50 °C in thermostated bath under magnetic stirring for 2 h. The supernatant was then filtered at 0.22 μm, appropriately diluted (1:10, 1:100, 1:200) with the mobile phase (A:B, 95:5, *v*/*v*, vide infra) and injected (20 μL) in triplicate for the analysis by HPLC-PDA-ESI-MS/MS.

### 3.4. HPLC-PDA-ESI-MS/MS Analysis

The aqueous extracts were analyzed with a Waters 1525 μ HPLC (Milford, MA, USA) equipped with a Waters 996 PDA detector and a Quattro Micro Tandem MS/MS with a Waters ESI source (Micromass, Manchester, UK), by using a Waters XBridge C18 (150 × 2.1 mm i.d.) 5 μm analytical column. Milli-Q water/formic acid 5 mM (A) and acetonitrile/formic acid 5 mM (B) were used as mobile phase, flowing at 0.20 mL/min. A chromatographic separation method previously developed was applied for the analysis [43,61,62]. Briefly: 0–1 min, 5% B; 1–20 min, 16.5% B; 20–30 min, 40% B; 30–35 min, 60% B; 35–36 min, 80% B; 36–40 min, 80% B; 40–41 min, 5% B; 41–61 min, 5% B. The PDA detector recorded one UV–Vis spectrum per second in the range of 200–800 nm, resolution 1.2 nm. Mass spectral data were acquired in: (a) full scan in negative (ESI-) and positive (ESI+) ionization; (b) selected ion recording (SIR) mode in ESI-, by using separate acquisition channels for each different *m*/*z* monoisotopic value of the deprotonated forms [M-H]^−^, under the following source conditions, previously optimized [43]: capillary voltage 2.7 kV, cone voltage 27 V, source temperature 120 °C, desolvation temperature 350 °C, cone gas flow 40 L/h, desolvation gas flow 500 L/h, dwell cell value of 0.200 s. Data acquisition, data handling, and instrument control were performed by MassLynx Software 4.1 v (Data Handling System for Windows, Micromass, UK).

### 3.5. Tryptophan Calibration Curve by HPLC-ESI-MS/MS

The methanol stock solution containing 1.0 mg/mL tryptophan was diluted with the mobile phase (A:B, 95:5, *v*/*v*) until the final concentrations of 0.1, 0.2, 0.5, 1.0 and 5.0 mg/L. Each final solution was analyzed in triplicate (20 μL injected) by HPLC-ESI-MS/MS-SIR mode, by selecting 203 *m*/*z* for the tryptophan monoisotopic anion [M-H]^−^. The calibration curve was calculated with equal-weighted least-squares linear regression analysis of the SIR peak area against the standard nominal concentration; limit of detection (LOD) and quantitation (LOQ) were obtained as LOD = 3Sa/b and LOQ = 10Sa/b, respectively, where Sa and b are the estimated standard deviation and the slope of the analytical calibration function with a 95% confidence level, respectively [65].

### 3.6. Untargeted ESI-MS/MS Profile

The untargeted analysis of the aqueous extracts, degassed, filtered and diluted 1:10 with the mobile phase (A:B, 95:5, *v*/*v*) was carried out by direct infusion into the ESI source with an external syringe flowing at 5 μL/min. Full scan spectral data were acquired for 2 min in the mass range 80–800 Da, in both ESI- and ESI+, with cone voltage 27 and 24 V, respectively, ionization source temperature 100 °C, desolvation gas temperature 150 °C, cone gas flow 30 L/h, and desolvation gas flow 400 L/h. The signals were normalized to the highest one for a direct comparison among samples. Fragmentation data were acquired in daughter mode in ESI- and/or ESI+, using argon as collision gas, by selecting each *m*/*z* value evidenced in the full scan as precursor ion, in turn, and using collision energy (CE) in the range of 10–20 eV.

### 3.7. Statistical Analysis

All samples were analyzed in triplicate for quantitation, and results were reported as mean values ± standard deviation (SD). Data were analyzed by using the one-way analysis of variance (ANOVA). The significance of differences (*p* < 0.05) among samples was determined by the Tukey test.

## 4. Conclusions

In conclusion, a comprehensive study of the *Agropyron repens* (L.) P. Beauv. rhizome herbal tea was carried out by ESI tandem–mass spectrometry, analyzing three independent commercial samples. ESI- and ESI+ mass spectra, acquired by direct infusion experiments, provided characteristic “rhizome” ions, ascribed to fructose/glucose, a di-hexose, and a di-hexose–pentose as typical saccharides, pyruvic, lactic, phosphoric, 2-furoic, fumaric, malic, cis-aconitic and citric acids as typical small organic acids, and γ-aminobutyric acid, proline, valine, asparagine, histidine and arginine as typical amino acids, with useful application for fast and simple quality control analysis. The HPLC-PDA-ESI-MS/MS analysis evidenced the unexpected presence of tryptophan, never reported previously, with the amounts lying in the range of 13.01–203.66 μg/g of dry rhizome, with possible new perspectives of applications for *Agropyron repens* rhizome. In addition, the targeted analysis evidenced at least twenty phenolic compounds representative of the *Agropyron repens* rhizome, among which caffeoyl and feruloyl quinic esters and caffeic and coumaric acids were the most abundant. From a comparison with aqueous extracts at different temperatures, the herbal tea resulted in a good compromise of temperature and extraction time, with a total of extracted phenolic antioxidants in the range of 126.89–289.09 μg/g of rhizome.

## Figures and Tables

**Table 1:** Chemical constituents of *Agropyron repens* rhizome extracts (literature data) [33,34].

Class	Compounds	Quantitation	Extraction Solvent	Ref.
Polysaccharides	fructosan (triticin), inulin	~12%	water	[10,35]
Disaccharides	not identified		water	[35]
Monosaccharides	fructose, glucose, rhamnose	(17.1, 3.4, nd%)	water	[10,35]
Sugar alcohols	mannitol, inositol	nd	nr	[6,36]
Phenolic acids, free (and bound)	chlorogenic acid	nd	nr	[10]
	*p*-coumaric acid	87 (24) μg/g	water	[37]
	*p*-hydroxybenzoic acid	10 (55) μg/g	water	[37]
	vanillic acid	27 (11) μg/g	water	[37]
	ferulic acid	38 (12) μg/g	water	[37]
	vanillin	4 (17) μg/g	water	[37]
	*p*-hydroxybenzaldehyde	tr (4) μg/g	water	[37]
Flavonoids	tricin	nd	nr	[10,19,38]
	rutin			
	hyperoside			
	baicalein			
	quercetin			
	luteolin			
Tannins		5%	water	[10]
Antraquinones	emodin	0.06–0.2 μg/g	acetonitrile/water	[39]
	chrysophanol	0.05–0.2 μg/g		
	physcion	0.08–0.3 μg/g		
Si	silicon species	0.4%	water	[40]
Main Volatiles	palmitic acid	0.05%	steam distillation	[6,16]
	carvacrol			
	*trans*-anethole			
	carvone			
	thymol			
	menthol			
	menthone			
Vitamins	ascorbic acid	nd	nr	[10]
	β-carotene			
Organic acids	acetic acid	nd	water	[10,41]
	propionic acid			
	butyric acid			
	hexanoic acid			
	phenylacetic acid			
	succinic acid			
	cinnamic acid			
	*p*-hydroxyphenylpropionic acid			
	*p*-coumaric acid			
	3,4-dihydroxyphenylpropionic acid			
Total polyphenols contents		257.95 μgGAE/mL	ethanol/water	[11]
Total flavonoid contents		70.89 μgRE/mL	ethanol/water	[11]
Antioxidant activity		~35% (DPPH)	ethanol/water	[11]
		~60% (reducing power)	ethanol/water	[11]

nd: not determined; nr: not reported; GAE: gallic acid equivalent; RE: rutin equivalent; DPPH: 2,2-diphenyl-1-picrylhydrazyl; reducing power: with respect to Fe^3+^ to Fe^2+^.

**Table 2:** Plant material characterization: absolute humidity (Abs. H); active water at 21.5 °C (aw); total aerobic count (TAC); yeast and molds (Y&M); lactic acid bacteria (LAB); Enterobacteriaceae (EB); CFU: colony-forming unit.

Sample	Abs. H (g/m^3^)	aw (21.5 °C)	TAC (CFU/g)	Y&M (CFU/g)	LAB (CFU/g)	EB (CFU/g)
AR1	9.53	0.495 ± 0.001	1.41 ± 0.15 × 10^3^	18.0 ± 0.10	<10	<10
AR2	10.60	0.545 ± 0.001	3.05 ± 0.20 × 10^3^	10.0 ± 0.20	<10	<10
AR3	8.63	0.485 ± 0.001	3.83 ± 0.50 × 10^3^	22.00 ± 0.14	<10	<10

**Table 3:** Fragmentation data obtained by negative ESI-MS/MS infusion experiments in daughter scans for the precursor ions assigned to pyruvic (Pyr ac), lactic (Lac ac), phosphoric (Pho ac), 2-furoic (Fur ac), fumaric (Fum ac), malic (Mal ac), *cis*-aconitic (*cis*-Aco ac) and citric (Cit ac) acids.

Organic Acid	Precursor [M-H]^−^ (*m*/*z*)	Fragments (*m*/*z*)	Ref.
Pyr ac	87	43 [M-H-CO_2_]^−^	[52]
Lac ac	89	71 [M-H-H_2_O]^−^; 43 [M-H-HCO_2_H]^−^	[52]
Pho ac	97	79 [M-H_2_O]^−^	[53]
Fur ac	111	67 [M-H-CO_2_]^−^; 41 [M-H-CO_2_-C_2_H_2_]^−^	[54]
Fum ac	115	71 [M-H-CO_2_]^−^	[55]
Mal ac	133	115 [M-H-H_2_O]^−^; 89 [M-H-CO_2_]^−^; 71 [M-H-CO_2_-H_2_O]^−^	[55]
*cis*-Aco ac	173	111 [M-CO_2_-H_2_O]^−^	[56]
Cit ac	191	129 [M-H-CO_2_-H_2_O]^−^; 111 [M-H-CO_2_-2H_2_O]^−^	[55]

**Table 4:** Fragmentation data obtained by positive ESI-MS/MS infusion experiments in daughter scans for the precursor ions assigned to γ-aminobutyric acid (GABA), proline (Pro), valine (Val), asparagine (Asn), histidine (His) and arginine (Arg).

Amino Acid	Precursor [M+H]^+^ (*m*/*z*)	Fragments (*m*/*z*)	Ref.
GABA	104	87	[58]
		69	[59]
Pro	116	70 [C_4_H_8_N]^+^	[60]
Val	118	72 [M+H-HCO_2_H]^+^	[60]
Asn	133	87 [M+H-HCO_2_H]^+^; 74 [NH_2_=CHCO_2_H]^+^	[60]
His	156	110 [M+H-HCO_2_H]^+^	[60]
Arg	175	116 [M+H-NH_2_C(NH)=NH_2_]^+^; 70 [C_4_H_8_N]^+^	[60]

**Table 5:** Qualitative and quantitate data of phenolic compounds and tryptophan, identified by comparison with standard, in AR1, AR2, AR3 herbal tea (IN), aqueous extracts at 50 °C (50) and 25 °C (25), reported as μg/g of dry rizhome, mean value of analysis in triplicate ± standard deviation (σ). t_R_: retention time; [M-H]^−^: monoisotopic mass of the deprotonated ion used for the SIR channel; values with different letters within rows are significantly different (*p* < 0.05): a, b, c refer to the same rhizome (AR1, AR2 or AR3) extracts at different temperatures; A, B, C, refer to the three herbal teas AR1, AR2 and AR3. nd: not detected; < LOD: under LOD, calculated from the corresponding calibration curve. AR1-IN, AR2-IN, AR3-IN: herbal tea; AR1-50, AR2-50, AR3-50: aqueous extracts at 50 °C; AR1-25, AR2-25, AR3-25: aqueous extracts at 25 °C. EA quantitated as quercetin equivalent; 3-FQA and 4-FQA tentatively assigned and quantitated as ferulic acid equivalent.

Compound	t_R_ (min)	AR1-IN (μg/g ± σ)	AR1-50 (μg/g ± σ)	AR1-25 (μg/g ± σ)	AR2-IN (μg/g ± σ)	AR2-50 (μg/g ± σ)	AR2-25 (μg/g ± σ)	AR3-IN (μg/g ± σ)	AR3-50 (μg/g ± σ)	AR3-25 (μg/g ± σ)	[M-H]^−^ (*m*/*z*)
GA	3.15	0.70 ± 0.08 ^a,A^	2.78 ± 0.08 ^b^	0.63 ± 0.04 ^a^	0.67 ± 0.09 ^a,A^	0.60 ± 0.08 ^a^	0.61 ± 0.07 ^a^	2.80 ± 0.13 ^a,B^	1.90 ± 0.25 ^a^	5.10 ± 0.00 ^b^	169
3,5-DHBA	4.61	nd	nd	nd	nd	nd	nd	nd	nd	nd	153
PCA	5.15	1.49 ± 0.19 ^a,A^	6.64 ± 0.25 ^b^	2.99 ± 0.12 ^c^	1.21 ± 0.09 ^a,A^	1.85 ± 0.08 ^a^	2.03 ± 0.05 ^b^	7.16 ± 0.56 ^a,B^	6.19 ± 0.19 ^a^	7.39 ± 0.92 ^a^	153
TP	5.51	19.92 ± 1.19 ^a,A^	20.52 ± 1.92 ^a^	12.61 ± 0.41 ^b^	203.66 ± 18.70 ^a,B^	116.20 ± 18.64 ^b^	196.05 ± 3.30 ^c^	13.01 ± 0.63 ^a,C^	20.76 ± 3.89 ^b^	20.99 ± 2.13 ^b^	203
5-CQA	6.90	57.40 ± 4.40 ^a,A^	59.02 ± 5.08 ^a^	20.06 ± 0.60 ^b^	107.56 ± 7.04 ^a,B^	101.23 ± 0.59 ^b^	105.10 ± 6.02 ^b^	112.22 ± 5.96 ^a,C^	220.06 ± 22.42 ^b^	202.31 ± 26.06 ^b^	353
GeA	7.14	0.86 ± 0.02 ^a,A^	2.38 ± 0.03 ^b^	0.87 ± 0.06 ^a^	1.23 ± 0.06 ^a,B^	1.10 ± 0.09 ^a^	1.63 ± 0.19 ^b^	2.07 ± 0.27 ^a,B^	4.19 ± 0.34 ^a^	3.46 ± 0.34 ^b^	153
Ca	7.32	nd	nd	nd	nd	nd	nd	nd	nd	nd	289
4-CQA	7.55	32.43 ± 0.65 ^a,A^	35.69 ± 0.64 ^b^	17.45 ± 1.75 ^c^	65.66 ± 3.92 ^a,B^	47.09 ± 1.56 ^b^	54.34 ± 6.24 ^b^	46.39 ± 4.75 ^a,C^	85.84 ± 2.55 ^b^	74.88 ± 8.81 ^c^	353
*p*-HBA	7.73	5.32 ± 0.47 ^a,A^	3.01 ± 0.78 ^b^	4.82 ± 0.59 ^a^	3.64 ± 0.47 ^a,B^	6.04 ± 0.57 ^b^	4.69 ± 0.82 ^a^	9.52 ± 0.94 ^a,C^	8.73 ± 0.55 ^b^	10.67 ± 1.00 ^b^	137
3-FQA	7.77	9.99 ± 0.83 ^a,A^	53.70 ± 3.42 ^b^	13.03 ± 0.04 ^c^	53.99 ± 0.18 ^a,B^	33.43 ± 0.79 ^b^	65.19 ± 4.89 ^c^	16.08 ± 0.25 ^a,C^	25.01 ± 3.84 ^b^	56.21 ± 2.75 ^c^	367
VA	9.13	3.18 ± 0.24 ^a,A^	6.37 ± 0.19 ^b^	2.60 ± 0.21 ^c^	3.36 ± 0.24 ^a,A^	4.07 ± 0.36 ^b^	4.39 ± 0.42 ^b^	5.22 ± 0.54 ^a,B^	6.91 ± 0.59 ^b^	8.87 ± 0.38 ^c^	167
CA	9.45	2.55 ± 0.14 ^a,A^	34.88 ± 0.30 ^b^	16.57 ± 2.54 ^c^	6.98 ± 0.34 ^a,B^	25.90 ± 1.69 ^b^	12.13 ± 1.29 ^c^	6.91 ± 0.59 ^a,B^	26.86 ± 2.21 ^b^	86.52 ± 2.09 ^c^	179
SyA	9.56	1.89 ± 0.26 ^a,A^	3.25 ± 0.40 ^b^	1.76 ± 0.08 ^a^	1.95 ± 0.20 ^a,A^	1.77 ± 0.06 ^b^	2.20 ± 0.11 ^a^	3.69 ± 0.20 ^a,B^	4.59 ± 0.29 ^b^	4.80 ± 0.44 ^b^	197
2,6-DHBA	10.48	<LOD	1.60 ± 0.06	<LOD	0.85 ± 0.11 ^a,A^	1.58 ± 0.12 ^b^	1.35 ± 0.06 ^b^	1.02 ± 0.08 ^a,A^	2.61 ± 0.05 ^b^	2.42 ± 0.16 ^b^	153
m-HBA	10.66	nd	nd	nd	nd	nd	nd	nd	nd	nd	137
4-FQA	12.12	3.97 ± 0.35 ^a,A^	26.50 ± 1.37 ^b^	5.14 ± 0.38 ^c^	27.14 ± 2.82 ^a,B^	17.59 ± 0.06 ^b^	30.30 ± 2.97 ^a^	7.97 ± 0.18 ^a,C^	23.25 ± 3.52 ^b^	28.84 ± 3.23 ^c^	367
CouA	14.19	5.83 ± 0.56 ^a,A^	10.21 ± 1.41 ^b^	4.62 ± 0.03 ^c^	8.53 ± 0.27 ^a,B^	14.58 ± 0.40 ^b^	10.24 ± 0.67 ^c^	7.60 ± 0.08 ^a,C^	11.92 ± 1.38 ^b^	20.74 ± 1.94 ^c^	163
EA	15.74	<LOD	<LOD	<LOD	<LOD	0.18 ± 0.02 ^a^	1.24 ± 0.08 ^b^	<LOD	nd	nd	301
SiA	15.92	0.40 ± 0.06 ^a,A^	0.73 ± 0.11 ^b^	0.41 ± 0.11 ^a^	0.50 ± 0.03 ^a,A^	0.54 ± 0.04 ^a^	0.69 ± 0.10 ^a^	0.78 ± 0.03 ^a,B^	1.18 ± 0.10 ^b^	0.93 ± 0.09 ^b^	223
FA	16.13	1.97 ± 0.08 ^a,A^	7.55 ± 0.56 ^b^	2.36 ± 0.99 ^c^	3.58 ± 0.47 ^a,B^	11.13 ± 0.01 ^b^	6.29 ± 0.18 ^c^	4.05 ± 0.20 ^a,B^	8.28 ± 0.87 ^b^	13.98 ± 0.79 ^c^	193
My	16.74	nd	nd	nd	nd	nd	0.73 ± 0.07	nd	nd	nd	463
Ru	16.83	nd	nd	nd	nd	nd	nd	nd	nd	nd	609
Q-3-G	17.75	nd	<LOD	<LOD	<LOD	0.59 ± 0.03 ^a^	0.74 ± 0.06 ^b^	nd	nd	0.73 ± 0.03	463
K-3-R	20.07	nd	nd	nd	<LOD	<LOD	0.16 ± 0.02	nd	<LOD	nd	593
SaA	20.33	0.30 ± 0.04 ^a,A^	0.43 ± 0.03 ^a^	0.34 ± 0.05 ^a^	0.29 ± 0.52 ^a,A^	0.55 ± 0.08 ^b^	1.08 ± 0.11 ^c^	1.37 ± 0.15 ^a,B^	1.94 ± 0.19 ^b^	3.44 ± 0.17 ^c^	137
He	23.26	1.37 ± 0.22 ^a,A^	1.01 ± 0.02 ^a^	1.05 ± 0.04 ^a^	1.71 ± 0.28 ^a,B^	1.30 ± 0.07 ^b^	1.17 ± 0.12 ^b^	4.40 ± 0.58 ^a,C^	1.11 ± 0.11 ^b^	6.91 ± 0.58 ^c^	609
Q	27.76	<LOD	0.19 ± 0.01	<LOD	<LOD	0.16 ± 0.06 ^a^	1.66 ± 0.15 ^b^	<LOD	nd	<LOD	301
K	30.27	<LOD	<LOD	<LOD	<LOD	<LOD	0.42 ± 0.12	<LOD	<LOD	<LOD	285
i-XH	32.70	0.22 ± 0.005 ^a,A^	0.23 ± 0.002 ^a^	0.25 ± 0.01 ^a^	0.24 ± 0.005 ^a,A^	0.28 ± 0.004 ^a^	0.49 ± 0.12 ^b^	nd	nd	nd	353
XH	38.04	<LOD	<LOD	<LOD	<LOD	0.24 ± 0.02 ^a^	4.14 ± 0.21 ^b^	nd	nd	nd	353

## Data Availability

Not applicable.

## Sample Availability

Samples of the analyzed compounds are available from the authors.
